# Prognostic Impact of the Number of Examined Lymph Nodes in Stage II Colorectal Adenocarcinoma: A Retrospective Study

**DOI:** 10.1155/2020/8065972

**Published:** 2020-06-24

**Authors:** Purun Lei, Ying Ruan, Jianpei Liu, Qixian Zhang, Xiao Tang, Juekun Wu

**Affiliations:** ^1^Department of Gastrointestinal Surgery, The Third Affiliated Hospital, Sun Yat-sen University, Guangzhou, China; ^2^Department of Thyroid and Breast Surgery, The Third Affiliated Hospital, Sun Yat-sen University, Guangzhou, China; ^3^Medical Record Management Section, The Third Affiliated Hospital, Sun Yat-sen University, Guangzhou, China

## Abstract

**Background:**

Evaluation of lymph node status is critical in colorectal carcinoma (CRC) treatment. However, as patients with node involvement may be incorrectly classified into earlier stages if the examined lymph node (ELN) number is too small and escape adjuvant therapy, especially for stage II CRC. The aims of this study were to assess the impact of the ELN on the survival of patients with stage II colorectal cancer and to determine the optimal number.

**Methods:**

Data from the US Surveillance, Epidemiology, and End Results (SEER) database on stage II resected CRC (1988-2013) were extracted for mathematical modeling as ELN was available since 1988. Relationship between ELN count and stage migration and disease-specific survival was analyzed by using multivariable models. The series of the mean positive LNs, odds ratios (ORs), and hazard ratios (HRs) were fitted with a LOWESS (Locally Weighted Scatterplot Smoothing) smoother, and the structural break points were determined by the Chow test. An independent cohort of cases from 2014 was retrieved for validation in 5-year disease-specific survival (DSS).

**Results:**

An increased ELN count was associated with a higher possibility of metastasis LN detection (OR 1.010, CI 1.009-1.011, *p* < 0.001) and better DSS in LN negative patients (OR 0.976, CI 0.975-0.977, *p* < 0.001). The cut-off point analysis showed a threshold ELN count of 21 nodes (HR 0.692, CI 0.667-0.719, *p* < 0.001) and was validated with significantly better DSS in the SEER 2009 cohort CRC (OR 0.657, CI 0.522-0.827, *p* < 0.001). The cut-off value of the ELN count in site-specific surgeries was analyzed as 20 nodes in the right hemicolectomy (HR 0.674, CI 0.638-0.713, *p* < 0.001), 19 nodes in left hemicolectomy (HR 0.691, CI 0.639-0.749, *p* < 0.001), and 20 nodes in rectal resection patients (HR 0.671, CI 0.604-0.746, *p* < 0.001), respectively.

**Conclusions:**

A higher number of ELNs are associated with more-accurate node staging and better prognosis in stage II CRCs. We recommend that at least 21 lymph nodes be examined for accurate diagnosis of stage II colorectal cancer.

## 1. Introduction

Evaluation of lymph node (LN) status is critical for predicting the prognosis of patients who have undergone radical surgery for colorectal carcinoma (CRC), plays a vital role in precise nodal staging, and affects postoperative adjuvant therapies. It is well known that patients of stage III CRC have to undergo the subsequent chemo/radiotherapy after surgery. However, the necessity of adjuvant chemotherapy for stage II CRC (T3-T4 without LN or distant metastasis) is still under debate [1, 2]. One evaluating metric for the subsequent therapy is <12 examined lymph nodes (ELNs). The threshold, according to the American Joint Committee on Cancer (AJCC) guidelines, is the assessment of at least 12 LNs [3]. However, patients with LN involvement (stage III CRC) may be incorrectly classified into stages I or II if the number of ELNs is too small. These patients may escape subsequent adjuvant therapy and suffer the worsen prognosis. Recent articles reported that a higher number of ELNs were associated with better prognosis and demonstrated the value of a higher cut-off point in stage II CRC patients [4–7].

In order to tackle the controversies between guidelines and studies, we investigated the large population-based cohorts undergoing CRC resections, to provide a more robust conclusion on the association of ELN numbers with staging and survival in resected CRCs. The hypothesis was that a higher number of ELNs are associated with a higher possibility of involved LN detection and better prognosis among patients with stage II CRC.

## 2. Materials and Methods

### 2.1. Patient Population

Population-based data on CRC patients from the US Surveillance, Epidemiology, and End Results (SEER)-18 Program from 1988-2014 (https://seer.cancer.gov/) was extracted and evaluated, as ELN count was available since 1988. The inclusion criteria were patients with colorectal adenocarcinoma, who underwent surgical resection, a T3 or T4 tumor, tumor parameters available in the SEER-18 database, ELN numbers available, no distant metastasis, tumor-specific related death information was available.

Information about patients (sex, race, age, and year of diagnosis), tumors (site, stages, topography, morphology, and differentiation), treatments (resection type, chemotherapy, and radiotherapy), and outcome variables (follow-up time and survival status) were retrieved with the use of SEER∗Stat version 8.3.6 software for patients who fitted the criteria between 1988 and 2013. Furthermore, data for patients diagnosed stage III CRC, T3/T4 with LN metastasis was also extracted as a comparative group to analyze the relationship between ELN count and stage migration. An independent cohort of cases from 2014 was also retrieved for cut-off validation in 5-year disease-specific survival (DSS).

### 2.2. Statistical Analyses

We used the Chi-square test to compare differences between categorical variables and the *t*-test for continuous variables. The Cox proportional hazards regression model was used to determine the effect of the ELN number on disease-specific survival (DSS) and to visualize the survival curves, which were adjusted for other significant prognostic factors (age, gender, race, tumor site and stage, differentiation, radio/chemotherapy). Stage migration in the current study was defined as migration from N0 to N1/N2 of all T3/T4 patients of stage II and stage III CRC, without distant metastasis. Based on the assumption that more ELNs present a greater opportunity to identify positive LNs, stage migration was assessed by correlating the ELN number and node status (T3/T4 primary lesion, from no metastasis LN detection to metastasis LN detection) by using a binary logistic regression model after adjusting for other potential confounders (age, gender, race, tumor site and stage, differentiation). All calculations were performed with SPSS version 21.0 for Windows software (IBM Corporation, Chicago, IL, USA).

The curves of odds ratios (ORs; stage migration) and hazard ratios (HRs; DSS) of each ELN count compared with one ELN (as a reference) as well as the curves of the mean positive number and probability of undetected positive LNs were fitted by using a LOWESS (Locally Weighted Scatterplot Smoothing) smoother with a bandwidth of 2/3 (default) using R version 3.6.2 software (Bell Laboratories, Murray Hill, NJ, USA) as described by Liang [8]. Structural break points were determined by the Chow test with the use of SAS 9.3 software (SAS Institute, Cary, NC, USA) as described by Iyer et al. [9]. The break points were considered the threshold of clinical impact and *p* < 0.05 was considered statistically significant.

The current study is performed following the principles from the declaration of Helsinki. Patient was informed during the database enrollment individually. The study was approved by the institutional review board of the Third Affiliated Hospital of Sun-Yatsen University and uploaded to the SEER administration for database access application.

## 3. Results

### 3.1. Patient Characteristics and Distribution of the Examined Lymph Node Number

**Figure 1:**
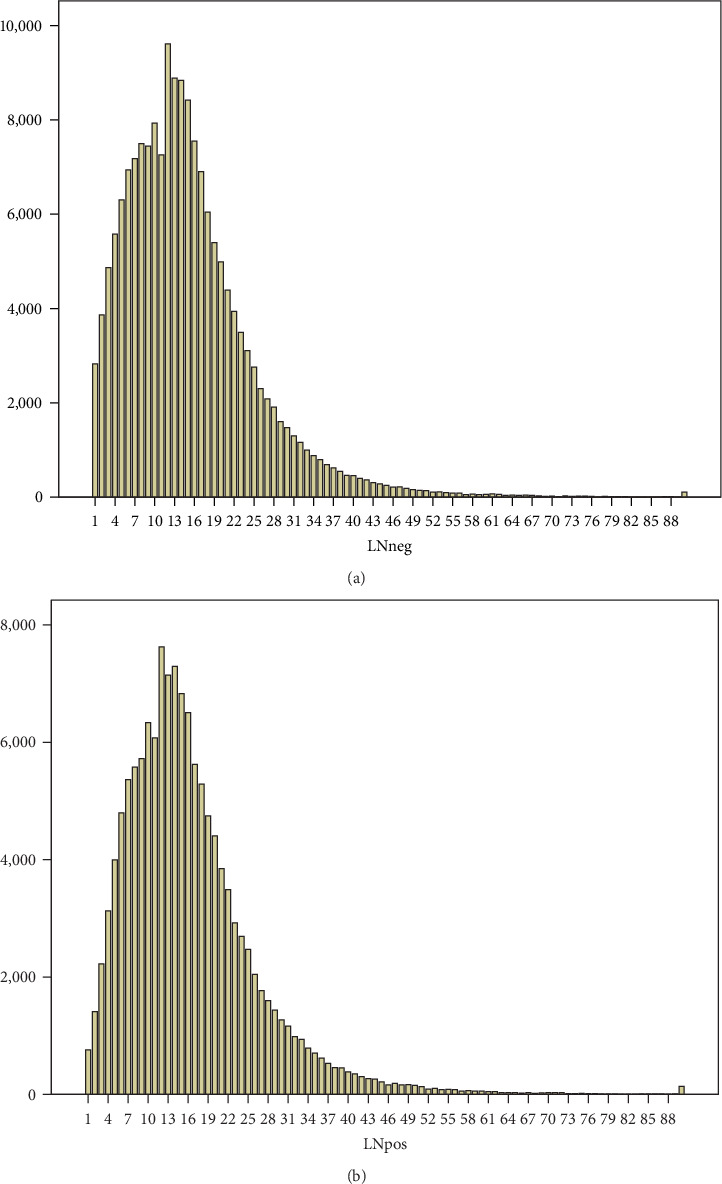
ELN number distribution of stage II (a) and III CRC (b).

173,355 patients from the SEER cohort with stage II CRC met the criteria and included into the main data analysis. Data for 135,130 patients who suffered from stage III with T3 or T4 was extracted in order to analyze the correlation between ELN count and LN positive incidence. An independent cohort of 4,410 cases from 2014 was retrieved for cut-off value validation. There were 29,082 tumor-specific deaths recorded (16.8%) during a median follow-up of 63 months in the SEER CRC stage II cohort, and 46,243 tumor-specific deaths recorded (34.22%) during a median follow-up of 43 months in the SEER CRC stage III (T3 and T4) cohort. The total tumor-specific deaths in the SEER 2014 CRC stage II validation cohort was 375 (8.5%). The baseline characteristics are shown in Table 1. The ELN distributions of stage II and III are shown in Figure 1.

### 3.2. Number of Examined Lymph Nodes and Population Stage Migration or Disease-Specific Survival

In order to clarify the correlation between the ELN volume and stage migration, patients' clinical data from the SEER database (1988-2013) LN negative, T3/T4, and SEER database (1988-2013) LN positive, T3/T4 groups were analyzed using the binary regression model. Poor differentiation (OR 1.680, CI 1.658-1.703, *p* < 0.001), T4 stage (OR 1.481, CI 1.452-1.509, *p* < 0.001), and larger ELN number (OR 1.010, CI 1.009-1.011, *p* < 0.001) were associated with a higher possibility of involved LN detection, while older age (OR 0.767, CI 0.756-0.779, *p* < 0.001) was the only protective factor for metastasis LN detection as shown in Table 2.

Furthermore, tumor-specific death analysis results showed that older age (OR 1.802, CI 1.759-1.848, *p* < 0.001), poor differentiation (OR 1.138, CI 1.113-1.163, *p* < 0.001), T4 stage (OR 2.120, CI 2.063-2.176, *p* < 0.001), and perioperative radiotherapy (OR 1.212, CI1.166-1.267, *p* < 0.001) were prognostic risk factors. However, female patients (OR 0.923, CI 0.902-0.945, *p* < 0.001), adjuvant chemotherapy (OR 0.844, CI 0.816-0.872, *p* < 0.001), and larger ELN count (OR 0.976, CI 0.975-0.977, *p* < 0.001) may help improve the long-term disease-specific survival of stage II CRC patients as shown in Table 2.

### 3.3. Cut-off Point Analysis for Patients with Node-Negative Disease and Validation

**Figure 2:**
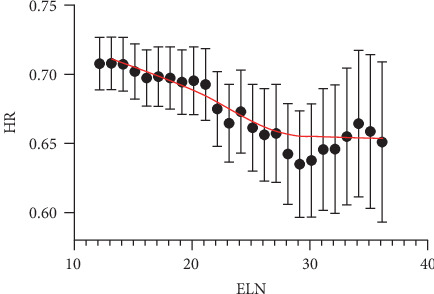
Fitting curves of ELN number HR of tumor-specific death in node-negative CRC.

**Figure 3:**
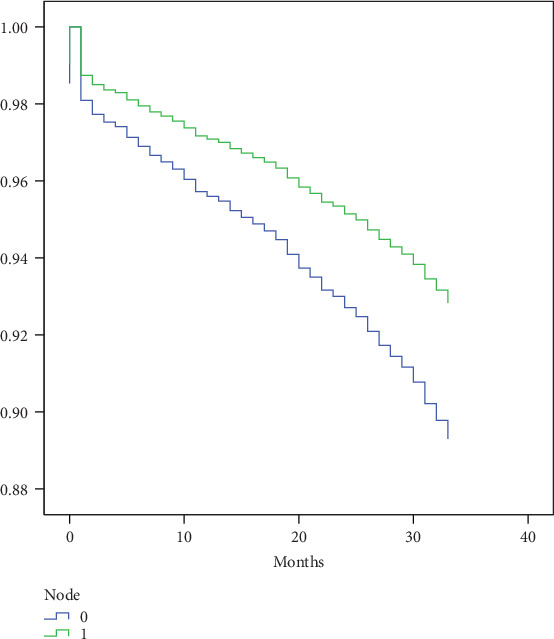
Validation K-M curves of SEER 2014 cohorts (green for ELN ≥21 nodes, blue for ELN <21 nodes).

Figure 2 shows the fitting curves of the ELN number hazard ratio of tumor-specific deaths in node-negative disease after controlling for other prognostic factors (gender, age, tumor location, differentiation grade, tumor stage, chemotherapy, and radiotherapy). The structural break point was estimated by the Chow test for probabilities of tumor-specific death. We found that 21 LNs were the critical cut-off point. The threshold was then validated in SEER 2014 cohorts, demonstrating significantly reduced tumor-specific deaths of patients with at least 21 LNs harvested in node-negative CRC (OR 0.657, CI 0.522-0.827, *p* < 0.001), as shown in Figure 3.

### 3.4. Subgroup Analysis

**Figure 4:**
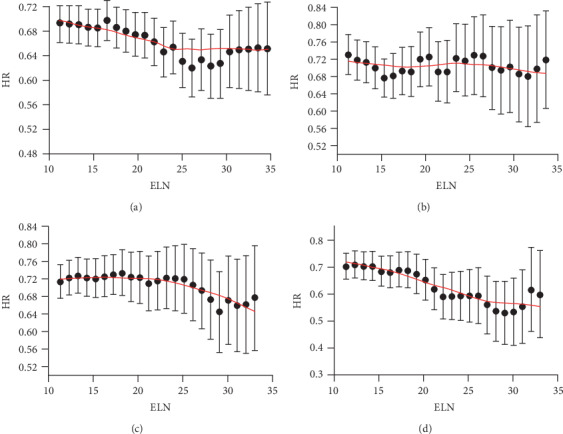
Fitting curves of right hemicolon resection (a), left hemicolon resection (b), sigmoid colon resection (c), and rectal resection (d).

Figure 4 shows the fitting curves of the ELN number HR of tumor-specific deaths in node-negative disease after controlling for other prognostic factors (gender, age, differentiation grade, tumor stage, chemotherapy, and radiotherapy) in tumor location subgroups such as the right hemicolon, left hemicolon, sigmoid colon, and rectum. The structural break point was estimated by the Chow test for probabilities of tumor-specific death. The ELN volume in site-specific surgeries was analyzed as 20 nodes in a right hemicolectomy, 19 nodes in a left hemicolectomy, and 20 nodes in rectal resection patients, respectively. However, sigmoid colon resections failed to definite a proper cut-off point.

## 4. Discussion

Examination of lymph nodes after radical colorectal surgery is an important parameter in CRC staging and could influence patients' long-term prognosis [10, 11]. Pathological T3/T4, without LN metastasis, was defined as stage II in the CRC staging system [3]. Once LN metastasis detected, tumor grade would upgrade into at least stage III. It is well known that patients of stage III CRC need adjuvant therapy and patients of stage II CRC without risk factors do not have to undergo adjuvant therapy [1, 2]; therefore, accurate staging plays a key role in CRC treatment.

As lymph node metastasis was the only difference in early and advanced CRCs, patients with lymph node involvement could be incorrectly classified into an earlier stage if the ELN number was too low. Such situations may lead to the adjuvant therapy remit and somehow worsen the prognosis. Thus, theoretically, a thorough examination of the surgical specimen is mandatory to assess the lymph node status of the tumor appropriately. Ideally, all the lymph nodes should be harvested from the surgical specimen and examined in order to confirm that a tumor is node-negative. At present, however, this goal is not practical. Various number of LNs in different colorectal regions and the extent of surgical lymphadenectomy influence the harvest LNs in the resection specimen. Furthermore, the diligence and skill of the pathologist in identifying and harvesting LNs determine the actual number of lymph nodes examined [12]. To tackle these intrinsic problems, a threshold of ELN is in great need in the CRC staging system.

Discussion on the ideal number ELN in CRC treatment has continued for decades. Scott [13] studied 50 cases of Dukes' C tumor and showed that 13 ELNs could cover 94% of all nodal positive patients. Hernanz [14] reported 6 LNs associated with 95% detection of at least 1 LN involved in Dukes' C CRC patients, while this probability increased to 99% if 10 LNs were examined. Tepper [15] divided patients with stage II rectal cancer into four quartiles according to the number of ELNs and suggested that 14 ELNs should be studied to define nodal status accurately. Cianchi [16] found that the 5-year survival rate of stage II patients with eight or fewer ELNs was similar to that of stage III patients, suggesting 9 ELNs might be sufficient for reliable staging of lymph node-negative tumors. Swanson [17] classified patients with T3N0 colon cancer into three groups according to the ELN and found that a minimum of 13 LNs should be examined. Destri [18] found that stage II patients with ELN <12 tend to have shorter DFS than stage II patients with ELN ≥12, and in stage III patients, ELN >12 is a better prognostic factor. These studies formed the guideline threshold of the AJCC recommendation of at least 12 LNs for all CRC patients [3]. However, most previous studies had important potential limitations, such as lack of adjustment for confounders, absence of stratified analyses, and limited sample size, resulting in limited robustness. Moreover, recently some articles focused on the ideal ELN in stage II CRCs. Tsai [4] reviewed a group of patients with stage I and II colorectal cancer and suggested that examining a minimum of 18 LNs per surgical specimen should be considered for more reliable staging of lymph node-negative cancer. Choi [5] found that 21 ELNs was an ideal cut-off value and was associated with better prognosis among 664 stage II CRC patients. Therefore, formulating the current hypothesis that there should be a better diagnostic value above 12 LNs in minimizing the false-negative incidence and improving the prognosis in stage II patients.

Stage migration regression results suggested that a larger number of ELNs were associated with a higher proportion of positive LN cases in the entire population, after adjusting for other risk factors such as age, gender, tumor location, differentiation, and T stage. Furthermore, the cohorts also exhibited a consistent positive correlation between a greater number of ELNs and better DSS in CRC with negative node status after adjusting for other risk factors such as age, gender, tumor location, differentiation, T stage, adjuvant chemotherapy, and radiotherapy. All these results demonstrated that a higher number of ELNs work as an independent factor in the detection of involved LNs and disease-specific prognosis in stage II CRC patients. There are some explanations for such phenomenon. As illustrated, the higher ELN numbers mean a thorough examination of the specimen that helps distinguish the metastasized LNs and results in stage migration. Secondly, a higher ELN count means a smaller chance of undiscovered positive LNs (malignancy remnants, the potential source of recurrence) and helps to accurately confirm the early CRC stage, which definitely has a better prognosis.

Correlation between higher ELN number between stage migration and DSS was confirmed; another key problem was an adequate threshold for the ELN count. The critical cut point would allow a confident postoperative claim of node-negative disease, rather than exhaustingly identifying maximal LNs in the specimen. Patients with fewer ELNs than the threshold might have a higher risk of residual positive LNs and poorer survival. Until now, the cut-off value was defined as at least 12 LNs in the AJCC and NCCN (National Comprehensive Cancer Network) guidelines [1–3]. However, the threshold was applied for all stages of CRCs aims to ensure a relatively satisfactory specimen evaluation based on the earlier articles. In the current study, we identified an optimal cut-off value of 21 ELNs for node-negative CRC (stage II CRC). The larger threshold could be considered one of the reference indexes for defining inadequate LN sampling for stage II CRC specimen. The current ELN count has been verified to have a protective prognostic impact among patients with node-negative disease in the 2014 cohort. The result suggests that at least 21 LNs should be harvested and examined for stage II CRC patients.

The prognosis for various CRC sites was different as recently reported due to histological inherent differences [19, 20]. A subgroup analysis was conducted according to the primary tumor location and surgical site. For the right hemicolon and rectal CRC, the ideal ELN volume was 20 LNs; for the left hemicolon, the ideal ELN was 20 LNs. These findings manifest a possibility that earlier CRC may somehow have the similar prognosis despite of the different primary sites. However, due to the primary data deficiency in the SEER-18 database as limited ELN number was bigger than 30, the current mathematical model failed to demonstrate an ideal threshold for the sigmoid colon. Nevertheless, the fitting curve of HRs begins to rise when the ELN number was bigger than 30 as shown in Figure 4. We believe that this cut-off point should be around 25 LNs. We believe future database supplementation in those bigger ELN numbers (>30 nodes) could help identify this threshold.

Lymph node collection from a colorectal resection specimen is time-consuming, particularly if the lymph nodes are small. Small lymph nodes are difficult to find especially amid large amounts of mesenteric fat. The diligence and skill of the pathologist in identifying and harvesting lymph nodes in the specimen determine the actual number of lymph nodes examined. It has been shown that nodal metastasis in colorectal cancer is often found in small lymph nodes (<5 mm in diameter) [21]. A diligent search for lymph nodes is required on gross examination of resection specimens. Our team researched a novel approach that may help a satisfactory LN dissection as methylene blue injection into IMA (inferior mesenteric artery) for rectal cancer right after specimen resection, especially for LN <5 mm [22]. But it had no influence on long-term survival. This may be due to the fact that the bias in patient selection without CRC stage stratification. The postoperative adjuvant therapy may also interfere the overall survival. Furthermore, instead of exhaustive lymph node harvest and examination, another alternative way to determine LN status is by a sentinel lymph node biopsy. This approach has been extensively used in breast cancer and melanoma. However, the value of a sentinel lymph node biopsy for colorectal cancer is still limited [23].

To our knowledge, this study is currently the largest one on these ELN evaluation issues that use multicenter, real-world data sets with robust statistics. We should emphasize two major points. First, the ELN count is associated with improved outcomes in CRC. Second, there is an increasing need to set up a specially cut-off point for stage II CRC or a novel node stage system in precise stratification [24].

This study is limited by its real-world retrospective nature. Surgical procedures, assessments, and enumerations of LNs varied among regions, surgeons, laboratories, and pathologists. We were not able to investigate some other important aspects such as the respective impact of the number of groups of LNs. We were unable to use uniform counting methods because of the population study design. We believe further larger multicenter with restricted criteria analysis may help eliminate these limitations.

## 5. Conclusion

In conclusion, a greater number of ELNs is associated with more-accurate node staging and better long-term survival in stage II CRCs. We recommend that at least 21 lymph nodes be examined for the accurate prognosis of stage II colorectal cancer.

## Figures and Tables

**Table 1:** Baseline characteristics of stage II, stage III, and validation cohort patients.

Cohort	SEER database (1988-2013) LN negative, T3/T4	SEER database (1988-2013) LN positive, T3/T4	SEER database (2014) stage II
Total	173355	135130	4410
Sex			
Male	86629	67315	2306
Female	86726	67815	2104
Age	70.11 ± 13.20	67.74 ± 13.84	67.09 ± 13.82
Race			
White	143911	108518	3495
Black	16227	14307	487
Others	13217	12305	428
Grade			
Well differentiation	13401	6531	302
Moderate differentiation	130450	89418	3494
Poor differentiation	27073	35813	510
Undifferentiatiated	2431	3368	104
Surgical site			
Right hemicolon resection	71173	54468	1729
Transeverse colon resection	16158	10306	399
Left hemicolon resection	28459	23833	744
Sigmoid colectomy	34229	28395	916
Rectal resection	23336	18128	649
Chemotherapy	42874	75358	1243
Radiotherapy	22126	19052	630
T stage			
T3	148701	107457	3744
T4	24654	27679	666
N stage			
N0	173335	0	4410
N1	0	84955	0
N2	0	50175	0
ELN volume	14 (1-90)	15 (1-90)	18 (1-90)
Follow-up	63 (0-348)	43 (0-347)	28 (0-25)
Tumor-specific death	29082	46243	375

**Table 2:** 

	Stage migration (T3/T4, N0 to N1/N2)				Tumor-specific deaths (LN negative patients)			
	Sig.	OR	LL	UL	Sig.	OR	LL	UL
Age >70 years old	<0.001	0.767	0.756	0.779	<0.001	1.802	1.756	1.848
Female gender	0.260	1.008	0.994	0.023	<0.001	0.923	0.902	0.945
Poorer differentiation	<0.001	1.680	1.658	1.703	<0.001	1.138	1.113	1.163
T4 stage	<0.001	1.481	1.452	1.509	<0.001	2.120	2.063	2.179
Larger ELN volume	<0.001	1.010	1.009	1.011	<0.001	0.976	0.975	0.977
Chemotherapy	NA	NA	NA	NA	<0.001	0.844	0.816	0.872
Radiotherapy	NA	NA	NA	NA	<0.001	1.215	1.166	1.267

## Data Availability

All data was retrieved from the US Surveillance, Epidemiology, and End Results (SEER)-18 Program database (https://seer.cancer.gov/).
